# Efficacy of D5F3 IHC for detecting ALK gene rearrangement in NSCLC patients: a systematic review and meta-analysis

**DOI:** 10.18632/oncotarget.11806

**Published:** 2016-09-01

**Authors:** Hu Ma, Wen-Xiu Yao, Lang Huang, Su-Han Jin, Da-Hai Liu, Yuan Liu, Xu Tian, Jin-Hui Tian, Jian-Guo Zhou

**Affiliations:** ^1^ Department of Oncology, Affiliated Hospital of Zunyi Medical University, Zunyi 563000, China; ^2^ Department of Oncology, Affiliated Cancer Hospital of Medical School, University of Electronic Science and Technology of China, Sichuan Cancer Hospital and Institute & Cancer, The Second People's Hospital of Sichuan Province, Chengdu, 610000, China; ^3^ Affiliated Stemmatological Hospital of Zunyi Medical University, Zunyi 563000, China; ^4^ Department of Pharmacology and Key Laboratory of Basic Pharmacology of Ministry of Education, Zunyi Medical University, Zunyi 563000, China; ^5^ Chongqing Cancer Hospital and Institute, Chongqing, 40030, China; ^6^ Evidence-Based Medicine Center of Lanzhou University, Lanzhou, 730000, China

**Keywords:** NSCLC, D5F3, ALK, diagnostic accuracy, meta-analysis

## Abstract

We conducted a pooled analysis comparing the efficacy of an immunohistochemistry (IHC) assay using the D5F3 antibody with that of fluorescence *in situ* hybridization (FISH) for detecting ALK gene rearrangement in NSCLC patients. A total of 32 studies involving 5805 samples were included in this review. Pooled sensitivity for D5F3 IHC was 0.97 (95%CI: 0.93-0.98), specificity was 0.99 (95%CI: 0.98-1.00), PLR was 119.20 (95%CI: 57.79-245.89), NLR was 0.03 (95%CI: 0.02-0.07), DOR was 3526.66 (95%CI: 1344.71-9249.03), and AUROC was 1.00 (95%CI: 0.99-1.00). Meta-regression revealed that specimen type was a source of heterogeneity for specificity, and specimen type and FISH signal distance were sources of heterogeneity in the joint model. Subgroup analysis revealed that sensitivity and specificity were higher when the FISH signal distance standard was ≥ 2 than when it was ≥ 1. Sensitivity was higher for tumor specimens than for cell specimens; specificity was higher for cell specimens than for tumor specimens. In conclusion, the D5F3 IHC assay was nearly as effective as FISH for detection of ALK gene rearrangement in NSCLC patients.

## INTRODUCTION

Lung cancer is one of the most frequency diagnosed and deadly cancers worldwide, and non-small cell lung cancer (NSCLC) accounts for more than 85% of lung cancer cases [1, 2]. Anaplastic lymphoma kinase (ALK) gene rearrangement is responsible for approximately 3%-5% of NSCLC cases [3, 4]. Studies have reported that ALK-tyrosine kinase inhibitors (ALK-TKIs) increase response rate [5–7] and progression-free survival times [8] in ALK fusion-positive NSCLC patients. NCCN guidelines thus recommend detection of ALK gene fusion in metastatic NSCLC, and the use of the ALK-TKI crizotinib as a first-line treatment in ALK-positive patients [9].

It is therefore crucial to assess the efficacy of different methods for detecting ALK rearrangement. At present, fluorescence *in situ* hybridization (FISH), polymerase chain reaction (PCR), and immunohistochemistry (IHC) are commonly used to detect ALK fusion. NCCN guidelines recommend FISH as the gold standard for detecting ALK fusion [10], but FISH is expensive and labor-intensive. Studies examining polymerase chain reaction (PCR) for detection of ALK rearrangement found that PCR had high diagnostic performance compared to FISH [11, 12]. However, PCR also resulted in a high false positive rate, suggesting that high-quality RNA is needed for this method [13]. Recently studies [14–16] have also examined the clinical use of IHC with D5F3, 5A4, and ALK1 antibodies, the most cost-effective method, for detecting ALK rearrangement. Jiang *et al*. [17] conducted a meta-analysis of the diagnostic operating characteristics of IHC and concluded that IHC assays using D5F3 and 5A4 antibodies reliably detected ALK rearrangement in NSCLC. However, this study did not examine the diagnostic value of IHC screening methods using the D5F3 antibody. Although the D5F3 antibody is commonly used in the clinical setting, its efficacy remains largely unknown; we therefore conducted a systematic review and meta-analysis to assess the diagnostic accuracy of the D5F3 antibody in detecting ALK rearrangement.

## RESULTS

### Selection of studies

**Figure 1:**
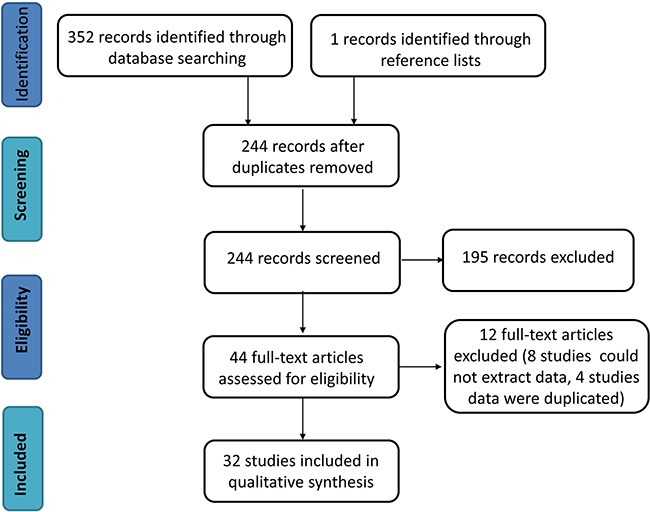
Flow chart of the systematic review process

A total of 352 literature citations were identified in database searches and 1 citation was identified from reference lists. Ultimately, 32 studies [12, 14, 15, 22–50] containing 37 trials and 5905 samples that met the inclusion criteria were included in this meta-analysis. 833 of these samples were positive and 4845 were negative for ALK gene rearrangement. Shen *et al*. [24] examined automated or manual detection of a D5F3 clone (Ventana) to detect ALK rearrangement; the rest of the studies used a different D5F3 clone (CST). Zhou *et al*. [12] used two difference samples, while Fu *et al*. [28] used EML4-ALK and ALK probes, to detect ALK rearrangement by FISH. Ying *et al*. [41] used two IHC positive standards. Figure 1 shows a flow diagram of the literature research process.

### Characteristics of included studies

All eligible studies were published between 2012 and 2015, and 19 of the studies were from China. Each study included one trial, with the following exceptions: Shen *et al*. [24] included 3 trials, Zhou *et al*. [12] involved 2 trails, Fu *et al*. [28] involved 2 parallel trials, and Ying *et al*. [41] included 2 trials. Of the 32 studies, 23 studies examined NSCLC specifically and 9 studies examined lung adenocarcinoma. Tumor tissues or cell blocks were used as FISH specimens, and details of the FISH and IHC procedures differed among the studies. The main characteristics of the included studies are shown in Supplementary Data 1.

### Quality assessment

**Figure 2:**
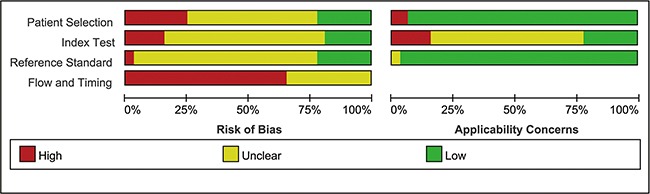
Risk of bias and applicability concerns summary

**Figure 3:**
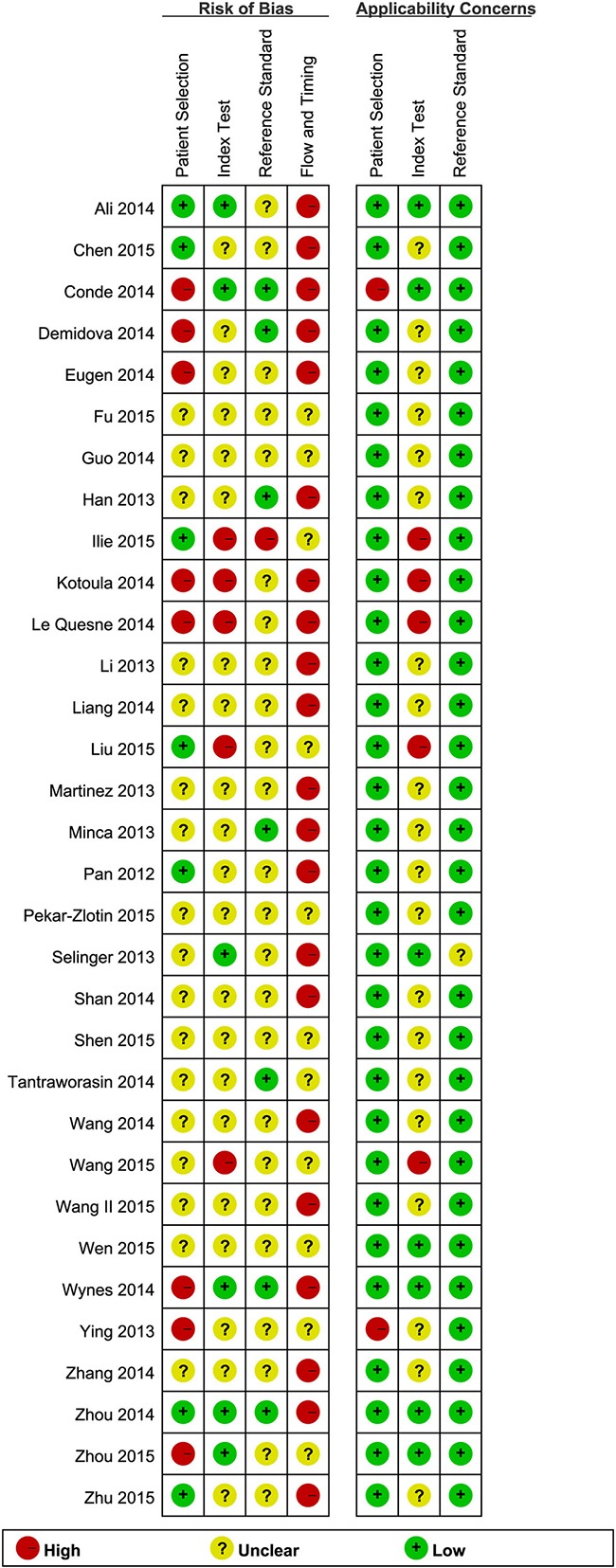
Risk of bias and applicability concerns graph

The methodological quality of the 32 studies was assessed using the QUADAS-2 tool. Risk of bias analysis revealed that 21 studies had high bias in flow and timing, 8 studies had high bias in patient selection, 5 studies had high bias in index tests, and 1 study had high bias in reference standard. Regarding applicability concerns, 31 studies had low bias in reference standard, 7 studies had low bias in index tests, and 30 studies had low bias in patient selection. Finally, the overall quality was acceptable. The details of methodological quality analysis of the included studies are summarized in Figures 2 and 3.

### Diagnostic performance

**Figure 4:**
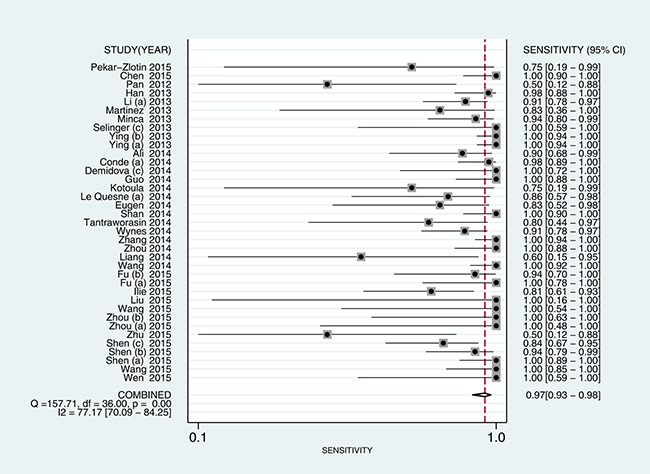
Forest plot estimating sensitivity of ALK rearrangement detection by D5F3 IHC in NSCLC patients in the selected studies Point estimates for sensitivity and 95% CIs are shown with pooled estimates; IHC = Immunohistochemistry; NSCLC = non-small cell lung cancer; ALK = anaplastic lymphoma kinase; CI = confidence interval; Q = Cochran Q statistic.

**Figure 5:**
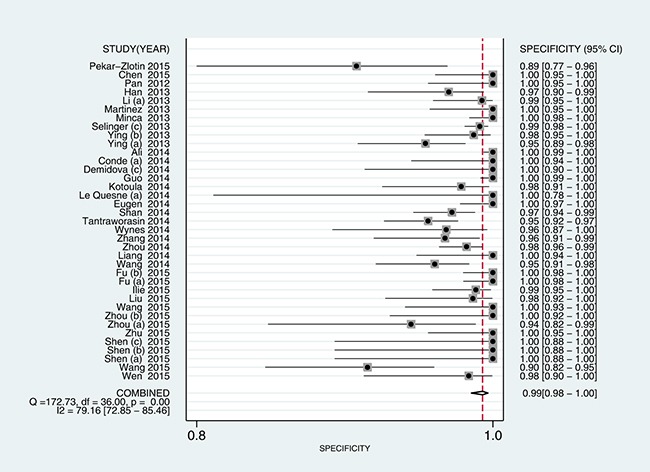
Forest plot estimating specificity of ALK rearrangement detection by D5F3 IHC in NSCLC patients in the selected studies Point estimates for specificity and 95% CIs are shown along with pooled estimates; IHC = Immunohistochemistry; NSCLC = non-small cell lung cancer; ALK = anaplastic lymphoma kinase; CI = confidence interval; Q = Cochran Q statistic.

**Figure 6:**
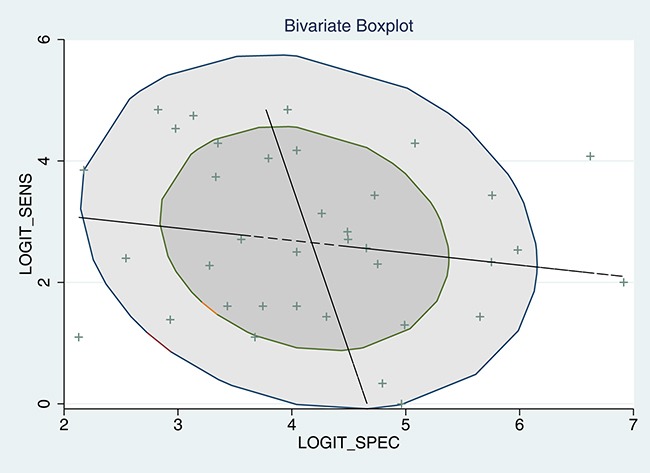
Bivariate boxplot of sensitivity and specificity in the 37 included trials

**Figure 7:**
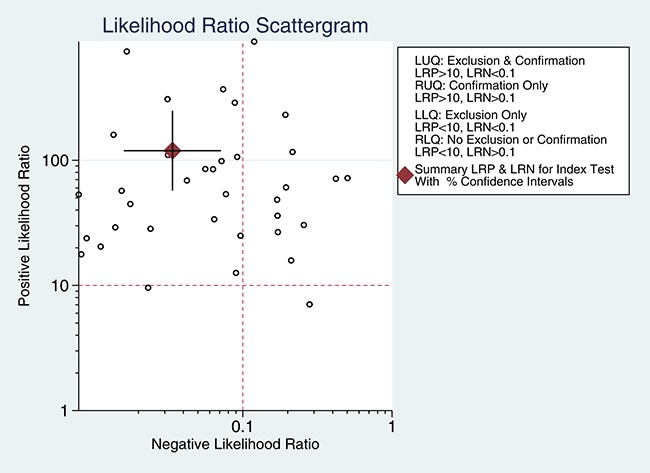
Likelihood ratio scattergram evaluating positive likelihood ratios ALK rearrangement detection with D5F3 IHC in NSCLC patients Point estimates for positive likelihood ratio and 95% CIs are shown along with pooled estimates; IHC = Immunohistochemistry; NSCLC = non-small cell lung cancer; ALK = anaplastic lymphoma kinase; CI = confidence interval.

**Figure 8:**
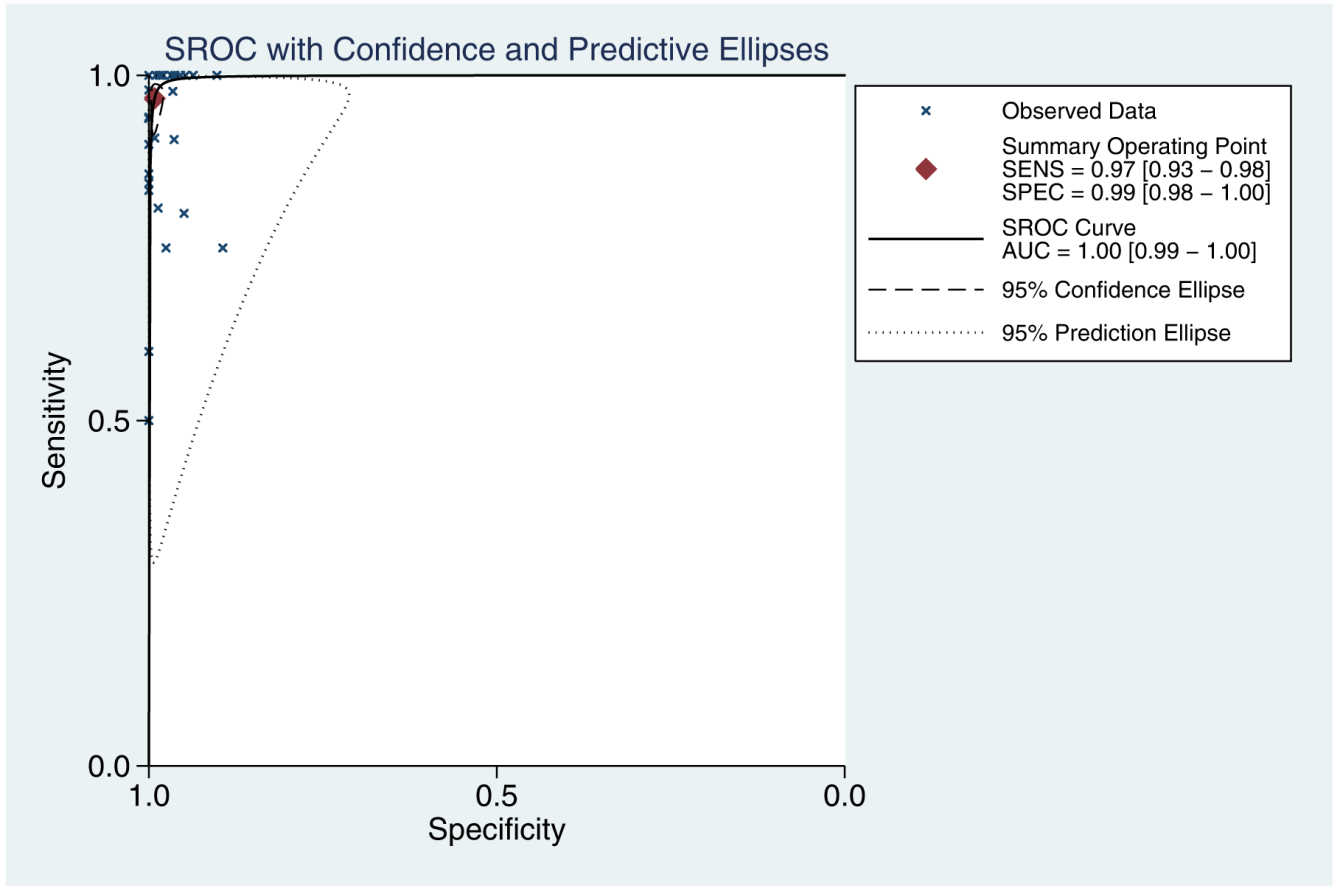
SROC curve for ALK rearrangement detection with the D5F3 IHC test in NSCLC patients AUC = area under the curve; IHC = Immunohistochemistry; NSCLC = non-small cell lung cancer; ALK = anaplastic lymphoma kinase; SROC = summary receiver-operating characteristic.

**Figure 9:**
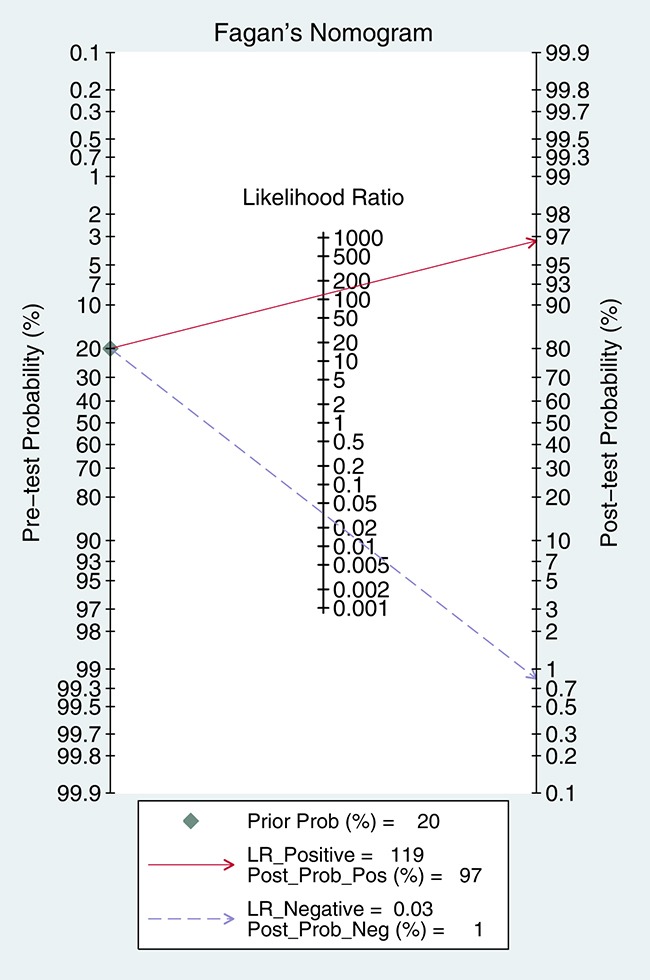
Fagan diagram evaluating overall diagnostic value of D5F3 IHC for ALK rearrangement detection in NSCLC patients IHC = Immunohistochemistry; NSCLC = non-small cell lung cancer; ALK = anaplastic lymphoma kinase; CI = confidence interval.

5905 samples were involved in this meta-analysis, and the overall pooled sensitivity of IHC for detection ALK fusion was 0.97 (95%CI: 0.93-0.98) (Figure 4), the specificity was 0.99 (95%CI: 0.98-1.00) (Figure 5), the PLR was 119.20 (95%CI: 57.79-245.89), the NLR was 0.03 (95%CI: 0.02-0.07), the DOR was 3526.66 (95%CI: 1344.71-9249.03), and the AUROC was 1.00 (95%CI: 0.99-1.00). Significant heterogeneity existed in this meta-analysis (Figure 6). As shown in Figure 7, the summary LRP and LRN for PCR was in the left upper quadrant (LUQ), indicating that the D5F3 IHC assay was a critical exclusion and confirmation method for detecting ALK fusion. The summary receiver operator characteristic (SROC) curve (Figure 8) indicated that the D5F3 IHC assay had high diagnostic performance in detecting ALK gene rearrangement; the corresponding area under the SROC curve (AUC) was 1.00 (95%CI:0.99-1.00). As shown in Figure 9, the clinical utility of D5F3 IHC for detecting ALK rearrangement was good, and the post-test probability (PLR: 97%, NLR: 1%) was greater than the pre-test probability (20%).

### Meta-regression analysis

The overall *I^2^* was 96.50, and the boxplot (Figure 6) showed that heterogeneity existed in the studies. Therefore, meta-regression was used to investigate potential sources of heterogeneity. Sample size, country, histological type, cells counted using FISH, FISH signal distance, supplier, manual or automated counting, specimen type, and IHC positive standard were included in the meta-regression analysis of sensitivity, specificity, and the joint model. Meta-regression results are shown in Table 1 and indicated that specimen type was a likely source of heterogeneity for specificity; specimen type and FISH signal distance were likely sources of heterogeneity for the joint model.

**Table 1:** Meta-regression results

**Table 1.1: Meta-regression of sensitivity**				
**Parameter**	**Estimate (95%CI)**	**Coef**	**Z**	***p* >|z|**
Sample size	0.97 [0.93 - 0.98]	3.34	0.01	0.99
Country	0.93 [0.81 - 0.97]	2.52	−1.71	0.09
Histological type	0.96 [0.89 - 0.99]	3.17	−0.41	0.68
FISH cells counted	0.95 [0.88 - 0.98]	3.04	−0.68	0.49
FISH signal distance	0.97 [0.93 - 0.98]	3.36	−0.13	0.90
Supplier	0.98 [0.92 - 0.99]	3.72	0.50	0.62
Manual or automated	0.96 [0.88 - 0.99]	3.20	−0.31	0.76
Specimen type	0.99 [0.94 - 1.00]	4.36	1.55	0.12
IHC positive standard I	0.96 [0.89 - 0.98]	3.11	−0.69	0.49
IHC positive standard II	0.94 [0.86 - 0.98]	2.84	−1.20	0.23
**Table 1.2: Meta-regression of specificity**				
**Parameter**	**Estimate (95%CI)**	**Coef**	**Z**	***p* >|z|**
Sample size	0.99 [0.98 - 1.00]	4.71	0.00	1.00
Country	0.99 [0.98 - 1.00]	4.90	0.19	0.85
Histological type	0.99 [0.97 - 1.00]	4.61	−0.49	0.62
FISH cells counted	0.99 [0.98 - 1.00]	5.23	0.90	0.37
FISH signal distance	0.99 [0.98 - 1.00]	4.88	1.75	0.08
Supplier	1.00 [0.98 - 1.00]	5.37	0.79	0.43
Manual or automated	0.99 [0.97 - 1.00]	4.58	−0.45	0.66
Specimen type	0.97 [0.94 - 0.99]	3.64	−2.47	0.01
IHC positive standard I	0.99 [0.98 - 1.00]	4.92	0.38	0.70
IHC positive standard II	0.99 [0.98 - 1.00]	5.01	0.49	0.62
**Table 1.3: Meta-regression of joint model**				
**Parameter**	**I^2^ (95%CI)**		**LRTChi**	***p* value**
Sample size	37.30 [0.00 - 100.00]		3.19	0.20
Country	41.67 [0.00 - 100.00]		3.43	0.18
Histological type	0.00 [0.00 - 100.00]		0.63	0.73
FISH cells counted	0.00 [0.00 - 100.00]		1.59	0.45
FISH signal distance	67.78 [27.85 - 100.00]		6.21	0.04
Supplier	9.21 [0.00 - 100.00]		2.20	0.33
Manual or automated	0.00 [0.00 - 100.00]		0.43	0.81
Specimen type	75.51 [46.28 – 100.00]		8.17	0.02
IHC positive standard I	0.00 [0.00 - 100.00]		0.61	0.74
IHC positive standard II	0.00 [0.00 - 100.00]		1.70	0.43

Sample size: >=150 vs. <150; Country: China vs. other countries; Histological type: lung adenocarcinoma vs. non-small cell lung cancer; FISH cells counted: >=50 vs. >=100; FISH signal distance: >=1 vs. >=2; Supplier: Ventana vs. other companies; Specimen type: tumor tissue vs. cell blocks; IHC Positive standard I: any percentage staining; IHC Positive standard II: any percentage staining or semi-quantitatively.

### Subgroup analysis

The results of subgroup analysis are shown in Table 2. Different FISH standards influenced the sensitivity and specificity of IHC. When FISH signal distance standard was ≥ 2, the sensitivity was 0.987 (95%CI: 0.983-0.991) and specificity was 0.983 (0.978- 0.987); when the standard was ≥ 1, the sensitivity was 0.952 (0.881-0.987) and the specificity was 0.963 (95%CI: 0.933-0.982). Regarding sample type, the sensitivity and specificity were 0.984 (95%CI: 0.960- 0.996) and 0.965 (95%CI: 0.951-0.976) for tumor samples and 0.936 (95%CI: 0.914- 0.954) and 0.987 (95%CI: 0.983-0.991) for cell samples, respectively. Finally, sensitivity and specificity were higher when the FISH standard was at least 2 than when it was at least 1. Additionally, sensitivity was higher for tumor specimens than for cell specimens, while specificity was higher for cells than for tumors.

**Table 2:** Subgroup analysis results about specimen type and FISH signal distance

Subgroup		Sensitivity	Specificity
Specimen type	Tumor	0.984 (0.960- 0.996)	0.965 (0.951-0.976)
	Cell	0.936 (0.914- 0.954)	0.987 (0.983-0.991)
FISH signal distance	>=1	0.952 (0.881-0.987)	0.963 (0.933-0.982)
	>=2	0.987 (0.983-0.991)	0.983 (0.978- 0.987)

### Publication bias

**Figure 10:**
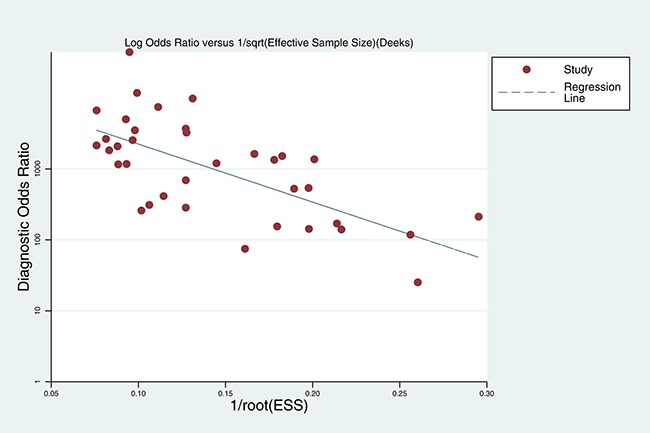
Deek's funnel plot evaluating publication bias

We used Deek's funnel plots of lnDOR against 1/ESS^1/2^, or, equivalently, against (1/*n*1 + 1/*n*2)^1/2^, which is proportional to 1/ESS^1/2^, to assess the accuracy of diagnostic tests [40]. The *p* value obtained from the funnel plot was 0.001, indicating the presence of publication bias in this meta-analysis (Figure 10).

### Level of evidence

TP, FP, TN, and FN were included in the Grade profile. The evidence qualities of TP and FN were moderate, and TN and FP were low. The evidence quality results are shown in Table 3.

**Table 3:** GRADE profile of evidence for included studies

Question: Should D5F3 IHC be used to diagnose ALK gene rearrangement in NSCLC patients?											
Sensitivity		0.97 (95% CI: 0.93 to 0.98)			Prevalences 5% 2% 7%						
Specificity		0.99 (95% CI: 0.98 to 1.00)									
Outcome	No of studies (No of patients)	Study design	Factors that may decrease quality of evidence					Effect per 1000 patients tested			Test accuracy QoE
			Risk of bias	Indirectness	Inconsistency	Imprecision	Publication bias	pre-test probability of 5%	pre-test probability of 2%	pre-test probability of 7%	
**True positives (patients** with **ALK Gene Rearrangement**)	32 studies5805 patients	cross-sectional (cohort type accuracy study)	not serious	not serious	not serious ^1^	not serious	publication bias strongly suspected ^2^	49 (47 to 49)	19 (19 to 20)	68 (65 to 69)	⨁⨁⨁○ MODERATE
**False negatives** (patients incorrectly classified as not having ALK gene rearrangement)								1 (1 to 3)	1 (0 to 1)	2 (1 to 5)	-
**True negatives** (patients without ALK gene rearrangement)	32 studies5805 patients	cross-sectional (cohort type accuracy study)	not serious	not serious	serious^3^	not serious	publication bias strongly suspected ^2^	941 (931 to 950)	970 (960 to 980)	921 (911 to 930)	⨁⨁○○ LOW
**False positives** (patients incorrectly classified as having ALK gene rearrangement)								9 (0 to 19)	10 (0 to 20)	9 (0 to 19)	-

1 I square is 66%

2 P =0.001

3 I square is 88%

## DISCUSSION

Siegel *et al*. first reported the presence of ALK rearrangement in NSCLC in 2007 [51]. While ALK fusion was previously found to occur in approximately 3%-5% of NSCLC patients [3, 4], we found ALK rearrangement rates of nearly 14%. This may be partially due to the large number of Chinese patients in our analysis; more studies are needed to examine possible differences among patient populations. Crizotinib and the newer ALK-TKIs ceritinib and alectinib are commonly used in clinical practice. While crizotinib improves progression-free survival (PFS) and response rates in NSCLC patients [52], almost all patients treated with crizotinib eventually experience progression. Ceritinib, with an overall response rate of 56% and median PFS of 7 months, is effective in ALK-positive metastatic NSCLC patients who progress during or are intolerant to crizotinib treatment [5]. Alectinib is also effective in those who progressed during crizotinib treatment, with a response rate of 50% and an 11.2-month median duration of response; additionally, it is effective for treating CNS disease [53].

FISH, IHC, and PCR are currently used to detect ALK gene fusion in NSCLC patients. FISH, a molecular diagnostic test, has been proved by the FDA for detecting ALK rearrangements and is regarded as the gold standard by most researchers. However, FISH is expensive and relatively labor-intensive. PCR, another method for detecting ALK fusion, is associated with high false positive rates, limiting its clinical utility. Some researchers have reported that IHC, a cost-effective and simple assay, can be used to screen for ALK rearrangements [40, 54], and three different antibodies are used for this purpose in clinical practice. D5F3, one of these antibodies, is widely used in clinics; we therefore conducted this systematic review and meta-analysis to assess the diagnostic accuracy of D5F3 IHC assays in detecting ALK rearrangement in NSCLC.

We examined 32 studies including 5805 samples among the 353 initially-identified literature citations in this study. High pooled sensitivity and specificity values indicated that D5F3 IHC had high diagnostic accuracy in detecting ALK rearrangement. Meanwhile, pooled PLR and NLR values further indicated high diagnostic accuracy for D5F3 IHC in clinical practice. Finally, pooled DOR and AUROC values indicated that D5F3 IHC had perfect discriminating ability. Although significant heterogeneity existed in our analysis, meta-regression revealed specimen type as a source of heterogeneity for specificity and specimen type and FISH signal distance as sources of heterogeneity for the joint model. Subgroup analysis revealed that sensitivity and specificity were higher when the FISH signal distance standard was ≥ 2 compared to ≥ 1. Finally, regarding specimen type, sensitivity was higher for tumor specimens than for cell specimens, while specificity was higher for cell specimens than for tumor specimens.

The present study expands upon the findings of Jiang *et al*. [17]. We searched more databases to identify studies and included more samples (5805 vs. 3754 patients), allowing our pooled analysis to more reliability evaluate the diagnostic value of IHC with the D5F3 antibody. Moreover, analysis with the QUADAS-2 tool indicated that the overall quality of the included studies was good. We also used the GRADE system to assess levels of evidence and meta-regression and subgroup analysis to investigate sources of heterogeneity in our meta-analysis.

However, the limitations of this study should also be considered when interpreting these results. First, the included studies used different standards for positive IHC and FISH results, possibly reducing the diagnostic accuracy. Second, the publication bias of the studies included in this investigation was significant. Moreover, our review only included studies published in English or Chinese; potentially relevant studies in other languages were excluded. Finally, although nonsmokers had a higher incidence of ALK gene rearrangement, we were unable to conduct subgroup analyses based on smoking status due to the limited availability of relevant information in the included studies.

In conclusion, our results indicate that IHC assays using the D5F3 antibody are nearly as effective as FISH in the detection of ALK gene rearrangement. Because IHC is more cost-effective and less labor-intensive than FISH, it might be a better method for primary ALK rearrangement screening in NSCLC patients.

## MATERIALS AND METHODS

Ethical approval and informed consent were not necessary for this meta-analysis study, which was conducted according to the guidelines of the *Cochrane Handbook for Diagnostic Test Accuracy Reviews*, available at http://srdta.cochrane.org. The protocol is registered with the Centre for Reviews and Dissemination PROSPERO database (available at: http://www.crd.york.ac.uk/PROSPERO/printPDF.php?RecordID=19905&UserID=7339, Registration No. CRD42015019905).

### Search strategy

A comprehensive literature search was conducted using the PubMed, EMBASE, Web of Science, Cochrane library, American Society of Clinical Oncology (ASCO), European Society for Medical Oncology (ESMO), China National Knowledge Infrastructure, China Wan Fang, and Chinese biomedical literature databases to identify studies published through March 2016. Search terms included anaplastic lymphoma kinase or ALK, immunohistochemistry or IHC, and fluorescence *in situ* hybridization or FISH. Only studies published in English or Chinese were examined. The reference lists of the reports selected in the original search were also examined to identify additional relevant studies. The PubMed search strategy is summarized in Supplementary Data 2.

### Study inclusion and exclusion criterion

Hu Ma and Lang Huang independently screened tiles, abstracts, and full texts, and disagreements were resolved by Jian-Guo Zhou. Eligible studies were required to meet the following criteria: (1) Patients were diagnosed with NSCLC; (2) D5F3 IHC assays were used to detect ALK fusion and compared to FISH; (3) Outcome data were presented in diagnostic 2×2 contingency tables (i.e., true positive [TP], false positive [FP], false negative [FN], and true negative [TN]); (4) IHC and FISH details were described; (5) Studies were designed as diagnostic tests. The exclusion criterion were as follows: (1) Reviews, meeting abstracts, or letters to the editor; (2) Insufficient data available; (3) Case reports or cohort studies.

### Data extraction and quality assessment

Hu Ma and Wen-Xiu Yao independently extracted the following information: study features (last name of the first author, year of publication, and country); number of samples; IHC and FISH details; type of specimen and outcome data (TP, FP, FN, and TN). The Quality Assessment of Diagnostic Accuracy Studies 2 (QUADAS-2) tool [18] and Review Manager 5.3 (The Nordic Cochrane Centre, The Cochrane Collaboration, 2014) were used to evaluate the methodological quality of selected studies. We assigned low, high, or unclear risk of bias values to the patient selection, index test, reference standard, and flow and timing domains; applicability concerns were also evaluated in the first three domains.

### Level of evidence

Jian-Guo Zhou used GRADEpro GDT (available at http://gdt.guidelinedevelopment.org/central_prod/_design/client/index.html), an all-in-one web solution for summarizing and presenting health care decision-making information, to evaluate level of evidence, and an evidence profile was generated to summarize the results. The GRADE system identified the following four rating grades of evidence quality [19]: High: further research is very unlikely to change our confidence in the effect estimate; Moderate: further research is likely to have an important impact on our confidence in the effect estimate and may change the estimate; Low: further research is very likely to have an important impact on our confidence in the effect estimate and is likely to change the estimate; and Very Low: any effect estimate is very uncertain.

### Statistical analysis

A bivariate regression model [20] was used to calculate the pooled sensitivity, specificity, PLR, NLR, DOR, and AUC and associated 95% confidence intervals (CIs). Bivariate boxplot, Chi-square, and inconsistency index (*I^2^*) were used to assess heterogeneity; an *I^2^* greater than 50% indicated significant heterogeneity [21]. Meta-regression and subgroup analysis were also used to investigate potential sources of heterogeneity. In addition, a likelihood ratio scatter gram was used to evaluate the exclusion and confirmation capacities of the index test. Finally, clinical utility and publication bias were assessed by a Fagan diagram and Deek's plot. STATA version 12.0 (Stata Corp, College Station, TX) was used for statistical analyses.

## SUPPLEMENTARY DATA






