# Supplementary material for: Efficacy of D5F3 IHC for detecting ALK gene rearrangement in NSCLC patients: a systematic review and meta-analysis
Source: Oncotarget. 2016 Sep 1;7(43):70128–42. doi: 10.18632/oncotarget.11806 (PMC5342540; doi:10.18632/oncotarget.11806)
Supplement: Supplementary file 1 [file oncotarget-07-70128-s001.pdf]

Efficacy of D5F3 IHC for detecting ALK gene rearrangement in NSCLC patients: a systematic review and meta-analysis

SUPPLEMENTARY DATA

Supplementary Data 1: PubMed search strategy.

See Supplementary File 1

Supplementary Data 2. Descriptive characteristics of included studies.

See Supplementary File 2
