# Supplementary material for: Efficacy of D5F3 IHC for detecting ALK gene rearrangement in NSCLC patients: a systematic review and meta-analysis
Source: Oncotarget. 2016 Sep 1;7(43):70128–42. doi: 10.18632/oncotarget.11806 (PMC5342540; doi:10.18632/oncotarget.11806)
Supplement: Supplementary file 2 [file oncotarget-07-70128-s002.docx]

PubMed Search List:

#1 ("Carcinoma, Non-Small-Cell Lung"[Mesh]) AND "anaplastic lymphoma kinase" [Supplementary Concept]) AND "In Situ Hybridization, Fluorescence" [Mesh]) AND "Immunohistochemistry"[Mesh])) OR (("Carcinoma, Non Small Cell Lung" OR "Carcinomas, Non-Small-Cell Lung" OR "Lung Carcinoma, Non-Small-Cell" OR "Lung Carcinomas, Non-Small-Cell" OR "Non-Small-Cell Lung Carcinomas" OR "Nonsmall Cell Lung Cancer" OR "Non-Small-Cell Lung Carcinoma" OR "Non Small Cell Lung Carcinoma" OR "Carcinoma, Non-Small Cell Lung" OR "Non-Small Cell Lung Cancer" OR "NSCLC")

#2 ("CD246 antigen" OR "anaplastic lymphoma receptor tyrosine kinase" OR "ALK tyrosine kinase receptor" OR "Alk protein, mouse" OR "anaplastic lymphoma receptor tyrosine kinase, mouse" OR "CD246 protein, mouse" OR "Tcrz protein, mouse" OR "anaplastic lymphoma kinase, mouse" OR "CD246 antigen, mouse" OR "Alk protein, rat" OR "anaplastic lymphoma receptor tyrosine kinase, rat" OR "anaplastic lymphoma kinase, rat" OR "ALK protein, human" OR "anaplastic lymphoma kinase Ki-1, human" OR "anaplastic lymphoma receptor tyrosine kinase, human" OR "CD246 antigen, human" OR "CD246 protein, human" OR "ALK tyrosine kinase receptor precursor protein, human" OR "anaplastic lymphoma kinase, human")

#3 ("Hybridization in Situ, Fluorescent" OR "FISH Technique" OR "Technique, FISH" OR "Techniques, FISH" OR "Fluorescent in Situ Hybridization" OR "FISH Technic" OR "FISH Technics" OR "Technic FISH" OR "Technics FISH" OR "Hybridization in Situ, Fluorescence" OR "In Situ Hybridization, Flurescent")

#4 ("Immunolabeling Techniques" OR "Immunolabeling Technique" OR "Technique, Immunolabeling" OR "Techniques, Immunolabeling" OR "Immunolabeling Technics" OR "Immunolabeling Technic" OR "Technic, Immunolabeling" OR "Technics, Immunolabeling" OR "Immunogold Techniques" OR "Immunogold Technique" OR "Technique, Immunogold" OR "Techniques, Immunogold" OR "Immunogold Technics" OR "Immunogold Technic" OR "Technic, Immunogold" OR "Technics, Immunogold" OR "Immunohistocytochemistry" OR "Immunogold-Silver Techniques" OR "Immunogold Silver Techniques" OR "Immunogold-Silver Technique" OR "Technique, Immunogold-Silver" OR "Techniques, Immunogold-Silver" OR "Immunogold-Silver Technics" OR "Immunogold Silver Technics" OR "Immunogold-Silver Technic" OR "Technic, Immunogold-Silver" OR "Technics, Immunogold-Silver" OR "Immunocytochemistry" OR "IHC")

#5 (#1 AND #2 AND #3 AND #4)
