# Supplementary material for: Efficacy of D5F3 IHC for detecting ALK gene rearrangement in NSCLC patients: a systematic review and meta-analysis
Source: Oncotarget. 2016 Sep 1;7(43):70128–42. doi: 10.18632/oncotarget.11806 (PMC5342540; doi:10.18632/oncotarget.11806)
Supplement: Supplementary file 3 [file oncotarget-07-70128-s003.docx]

| **Study** | **TP** | **FP** | **FN** | **TN** | **No. Analyzed** | **Country** | **tumor type** | **FISH** | **IHC** | **Type of specimen** | **IHC positive standard** |
| --- | --- | --- | --- | --- | --- | --- | --- | --- | --- | --- | --- |
| Wen 2015 | 7 | 1 | 0 | 52 | 60 | China | LUAD | Cell counted: 50 ＞15% Signal distance:＞1 Probe: ALK | Clone: D5F3 Suppier: Roche Manual or automated: automated | tumor tissue | any percentage |
| Wang 2015 | 23 | 9 | 0 | 83 | 115 | China | NSCLC | Cell counted: 50 ≥15% Signal distance:≥1 Probe : ALK | Clone: D5F3 Suppier: Cell Signaling Technology Manual or automated: manual | tumor tissue | any percentage |
| Shen (a) 2015 | 32 | 0 | 0 | 28 | 60 | China | NSCLC | Cell counted: 100 ＞15% Signal distance:＞2 Probe: ALK | Clone: D5F3 Suppier: Ventana Manual or automated: automated | tumor tissue | any percentage |
| Shen (b) 2015 | 30 | 0 | 2 | 28 | 60 | China | NSCLC | Cell counted: 100 ＞15% Signal distance:＞2 Probe: ALK | Clone: D5F3 Suppier: Ventana Manual or automated: manual | tumor tissue | any percentage |
| Shen (c) 2015 | 27 | 0 | 5 | 28 | 60 | China | NSCLC | Cell counted: 100 ＞15% Signal distance:＞2 Probe: ALK | Clone: D5F3 Suppier: Cell Signaling Technology Manual or automated: manual | tumor tissue | any percentage |
| Zhu 2015 | 3 | 0 | 3 | 71 | 77 | China | LUAD | Cell counted：- ＞15% Signal distance: ≥1 Probe: ALK | Clone: D5F3 Suppier: Cell Signaling Technology Manual or automated: manual | tumor tissue | >10% |
| Zhou (a) 2015 | 5 | 3 | 0 | 44 | 52 | China | NSCLC | Cell counted: 50 ≥15% Signal distance:≥2 Probe: ALK | Clone: D5F3 Suppier: Cell Signaling Technology Manual or automated: automated | Cell blocks | any percentage |
| Zhou (b) 2015 | 8 | 0 | 0 | 44 | 52 | China | NSCLC | Cell counted: 50 ≥15% Signal distance: ≥2 Probe: ALK | Clone: D5F3 Suppier: Cell Signaling Technology Manual or automated: automated | tumor tissue | any percentage |
| Wang 2015 | 6 | 0 | 0 | 52 | 58 | China | NSCLC | Cell counted: 100 ＞15% Signal distance: ≥2 Probe: ALK | Clone: D5F3 Suppier: Ventana Manual or automated: automated | Cell blocks | any percentage |
| Liu 2015 | 2 | 1 | 0 | 63 | 66 | China | NSCLC | Cell counted:100 ≥15% Signal distance: ＜2 and nature of 2p23 inversion Probe: ALK | Clone: D5F3 Suppier: Ventana Manual or automated: automated | Cell blocks | 1 |
| Ilie 2015 | 21 | 2 | 5 | 148 | 176 | France | LUAD | Cell counted:50 ≥15% Signal distance ≥1 Probe: ALK | Clone: D5F3 Suppier: Ventana Manual or automated: automated | tumor tissue | any percentage |
| Fu (a) 2015 | 15 | 0 | 0 | 158 | 173 | China | LUAD | Cell counted: ＞50 ＞15% Signal distance: ＞2 Probe: ALK | Clone: D5F3 Suppier: Cell Signaling Technology Manual or automated: manual | tumor tissue | >15% |
| Fu (b) 2015 | 15 | 0 | 1 | 157 | 173 | China | LUAD | Cell counted: ＞50 ＞15% Signal distance: ＞2 Probe: EML4-ALK | Clone: D5F3 Suppier: Cell Signaling Technology Manual or automated: manual | tumor tissue | >15% |
| Wang 2014 | 46 | 7 | 0 | 147 | 200 | China | LUAD | Cell counted: ≥100≥15% Signal distance: NR Probe: ALK | Clone: D5F3 Suppier: Cell Signaling Technology Manual or automated: automated | NR | any percentage |
| Liang 2014 | 3 | 0 | 2 | 60 | 65 | China | LUAD | Cell counted:50 ≥15% Signal distance: ＞ 2 Probe: ALK | Clone: D5F3 Suppier: Roche Diagnosis Manual or automated: automated | tumor tissue | any percentage |
| Zhou 2014 | 28 | 7 | 0 | 333 | 368 | China | LUAD | Cell counted: 50 ≥15% Signal distance: ≥2 Probe: ALK | Clone: D5F3 Suppier: Cell Signal Technology, Billerica Manual or automated: manual | tumor tissue | >15% |
| Zhang 2014 | 57 | 4 | 0 | 103 | 164 | China | NSCLC | Cell counted: 50 ≥15% Signal distance: ≥1 Probe: ALK | Clone: D5F3 Suppier: Cell Signal Technology Manual or automated: Manual | NR | 2 |
| Wynes 2014 | 39 | 2 | 4 | 53 | 98 | America | NSCLC | Cell counted: 50 ≥15% Signal distance: ≥1 Probe: ALK | Clone: D5F3 Suppier: Ventana Manual or automated: automated | Cell blocks and tumor tissue | any percentage |
| Tantraworasin 2014 | 8 | 13 | 2 | 244 | 267 | Thailand | NSCLC | Cell counted: 50 ＞15% Signal distance: ≥1 Probe: ALK | Clone: D5F3 Suppier: Ventana Manual or automated: automated | tumor tissue | 3 |
| Shan 2014 | 36 | 8 | 0 | 242 | 286 | China | LUAD | Cell counted: 50 ≥15% Signal distance: ≥2 Probe: ALK | Clone: D5F3 Suppier: Cell Signaling Technology Manual or automated: automated | NR | 4 |
| Eugen 2014 | 10 | 0 | 2 | 142 | 154 | America | NSCLC | Cell counted: 50 ≥15% Signal distance: NR Probe: ALK | Clone: D5F3 Suppier: Cell signaling Technology Manual or automated: automated | Cell blocks and tumor tissue | any percentage |
| Le Quesne (a) 2014 | 12 | 0 | 2 | 15 | 29 | England | NSCLC | Cell counted: 200 ≥15% Signal distance: NR Probe: ALK | Clone: D5F3 Suppier: Ventana Manual or automated: automated | Cell blocks and tumor ti*s*sue | 4 |
| Kotoula 2014 | 3 | 2 | 1 | 79 | 85 | Greece | NSCLC | Cell counted: NR ＞15% Signal distance: NR Probe: ALK | Clone: D5F3 Suppier: Cell Signaling Technology Manual or automated: maual | NR | ≥15% |
| Guo 2014 | 29 | 0 | 0 | 375 | 404 | China | LUAD | Cell counted: 50 ＞15% Signal distance: NR Probe: ALK | Clone: D5F3 Suppier: Roche/Ventana Manual or automated: automated | tumor tissue | any percentage |
| Demidova (c) 2014 | 11 | 0 | 0 | 35 | 46 | Russia | NSCLC | Cell counted: 50 ＞15% Signal distance: ≥2 Probe: ALK | Clone: D5F3 Suppier: Cell Signaling Technology Manual or automated: automated | tumor tissue | positive (3+ or 2+) or negative (1+ or 0) |
| Conde (a) 2014 | 46 | 0 | 1 | 56 | 103 | Italy | NSCLC | Cell counted: ≥50 ≥15% Signal distance: ≥1 Probe: ALK | Clone: D5F3 Suppier: Ventana Manual or automated: automated | NR | 5 |
| Ali 2014 | 18 | 0 | 2 | 503 | 523 | Italy | NSCLC | Cell counted: NR ≥15% Signal distance: NR Probe: ALK | Clone: D5F3 Suppier: Ventana Manual or automated: automated | tumor tissue | 6 |
| Ying (a) 2013 | 63 | 7 | 0 | 126 | 196 | China | LUAD | Cell counted: 50 ≥15% Signal distance: ≥1 Probe: ALK | Clone: D5F3 Suppier: Cell Signaling Technology Manual or automated: automated | tumor tissue | >=1 |
| Ying (b) 2013 | 63 | 2 | 0 | 131 | 196 | China | LUAD | Cell counted: 50 ≥15% Signal distance: ≥1 Probe: ALK | Clone: D5F3 Suppier: Ventana Manual or automated: automated | tumor tissue | any percentage |
| Selinger (c) 2013 | 7 | 6 | 0 | 581 | 594 | Australia | NSCLC | Cell counted: 50 ≥15% Signal distance: ≥2 Probe: ALK | Clone: D5F3 Suppier: Cell Signallign technology Manual or automated: automated | tumor tissue | any percentage |
| Minca 2013 | 31 | 0 | 2 | 198 | 231 | America | NSCLC | Cell counted: 50 ≥15% Signal distance: ≥1 Probe: ALK | Clone: D5F3 Suppier: Cell Signalling Technologies Manual or automated: automated | tumor tissue cell blocks | 6 |
| Martinez 2013 | 5 | 0 | 1 | 73 | 79 | America | NSCLC | Cell counted: ≥100 ＞5% Signal distance: NR Probe: ALK | Clone: D5F3 Suppier: Cell Signalling Technology Manual or automated: automated | tumor tissue and cell blocks | ≥10% |
| Li (a) 2013 | 40 | 1 | 4 | 116 | 161 | China | LUAD | Cell counted: 50 ＞15% Signal distance: 2 Probe: ALK | Clone: D5F3 Suppier: Cell Signaling Technology Manual or automated: automated | tumor tissue | 7 |
| Han 2013 | 42 | 3 | 1 | 84 | 130 | China | NSCLC | Cell counted: ≥50 ＞15% Signal distance: ≥2 Probe: ALK | Clone: D5F3 Suppier: Cell Signal Technology Manual or automated: manual | NR | 7 |
| Pan 2012 | 3 | 0 | 3 | 71 | 77 | China | LUAD | Cell counted: ≥50 ≥15% Signal distance: ≥2 Probe: ALK | Clone: D5F3 Suppier: Cell Signaling Technology Manual or automated: manual | tumor tissue | negative: 0 or 1+ positive 2+，3+ |
| Chen 2015 | 36 | 0 | 0 | 80 | 116 | China | NSCLC | Cell counted:50 ≥15% Signal distance: ≥2 Probe: ALK | Clone: D5F3 Suppier: Ventana Manual or automated: automated | tumor tissue and cell blocks | any percentage |
| Pekar-Zlotin 2015 | 3 | 5 | 1 | 42 | 51 | Israel | LUAD | Cell counted:50 ≥15% Signal distance: ≥1 Probe: ALK | Clone: D5F3 Suppier: Cell Signaling Technology Manual or automated: automated | NR | 8 |
| **LUAD**= lung adenocarcinoma;  **IHC positive standard 1=**0 for absent or barely perceptible expression, 1 (low) for weak to moderate multifocal expression, and 2 (high) for strong staining in most cells; **2**=HC results were scored as 0 when no specific staining was apparent within a tumor,1+, faint staining intensity in more than 10% tumor cells without any background staining,2+, moderate staining intensity,3+, strong staining intensity; **3**=negative result referred to none, mild or moderate positive cell staining; **4**= 0–5 (0 = 0%, 1≤1%, 2 = 1–10%, 3 = 11–33%, 4 = 34–66% and 5≥66%); **5**=positive (3+ or 2+) or negative (1+ or 0); **5**=Positive immunostaining for ALK was clearly visible as strong,granular, cytoplasmic staining in tumor cells; **6**= ALK IHC was scored using the scoring scheme proposed as follows: 0, no staining; 1+, faint cytoplasmic reactivity without any background staining; 2+, moderate cytoplasmic reactivity; and 3+, granular cytoplasmic reactivity of strong intensity in ≥10% of tumor cells.; **7**= scored as 3+ (strong staining), 2+ (moderate staining), 1+ (faint staining) without any background staining or 0, no staining at all. Scores of 0 and 1+ were considered negative and scores of 2+ and 3+ were considered positive for ALK overexpression; **8**= A positive result was defined as any positive staining considered to be specific on an intensity scale from 0 to 3,although an H-score system (0–300) was used for the final report. | | | | | | | | | | | |
